# Implementation Science Study Evaluating the Real‐World Use of Blood‐Based Biomarkers as Confirmatory Diagnostic Tools for Alzheimer's Disease in the United States

**DOI:** 10.1002/alz70856_099804

**Published:** 2025-12-25

**Authors:** Daryl Jones, Jo Vandercappellen, Leah Zullig, Marwan N. Sabbagh, Michelle M Mielke, Douglas R. Galasko, Nicholas Giordano, Christine Divers, Feride H Frech, Soeren Mattke, Harald Hampel, Hideki Toyosaki

**Affiliations:** ^1^ Eisai Inc., Nutley, NJ, USA; ^2^ Duke University School of Medicine, Durham, NC, USA; ^3^ Barrow Neurological Institute, Phoenix, AZ, USA; ^4^ Division of Public Health Sciences, Wake Forest University, School of Medicine, Winston‐Salem, NC, USA; ^5^ Shiley‐Marcos Alzheimer's Disease Research Center, University of California, San Diego, CA, USA; ^6^ PINC AI™ Applied Sciences, Premier, Inc, Charlotte, NC, USA; ^7^ University of Southern California, Los Angeles, CA, USA

## Abstract

**Background:**

High‐accuracy blood‐based biomarkers (BBMs) may allow accessible, scalable confirmation of amyloid pathology in the Alzheimer's disease (AD) care pathway. This study will investigate the feasibility of implementing BBMs as confirmatory diagnostic tools for AD in routine patient care.

The study's primary objective is to assess provider and health systems’ acceptability and appropriateness of using confirmatory BBMs in real‐world clinical practice, and explore healthcare systems' perceived barriers and facilitators for implementing confirmatory BBMs in real‐world clinical practice utilizing a Consolidated Framework for Implementation Research (CFIR).

**Method:**

This is a multiple methods, prospective, multi‐site implementation science study. We will survey up to 20 healthcare providers, administrative and support personnel from three clinics via electronic surveys (up to 20 participants), and conduct semi‐structured interviews (11 participants) and focus groups (up to 20 participants). Interviews and focus group data will use rapid qualitative analysis methods.

A CFIR was created to systematically identify and explore the complex factors that can influence the successful implementation of BBMs in the diagnosis of AD. The CFIR will be used as an organizing theoretical structure in this study. The protocol has received a central institutional review board approval.

**Result:**

This analysis will report quantitative and qualitative data from surveys and interviews, respectively. Clinician acceptability and appropriateness, and perceived barriers and facilitators for the implementation of confirmatory BBMs will be reported. The survey will collect Feasibility of Intervention Measure, Acceptability of Intervention Measure and Intervention Appropriateness Measure data; these are often considered predictors of implementation success. Participant characteristics (eg, age, sex, years in practice, credentials) and additional constructs that aim to explain and predict clinical practice changes will also be collected. Interviews will be mapped to relevant CFIR domains and constructs.

**Conclusion:**

Findings will provide insights into the feasibility of and potential barriers to implementing confirmatory BBMs into routine care. Evaluating the use of BBMs in real‐world settings may lead to efficient implementation at the population level, simplifying the AD patient care pathway by providing clinicians with a minimally invasive and non‐ionizing diagnostic for AD.

